# Optimizing the prediction of sepsis-associated encephalopathy with cerebral circulation time utilizing a nomogram: a pilot study in the intensive care unit

**DOI:** 10.3389/fneur.2023.1303075

**Published:** 2024-01-11

**Authors:** Jiangjun Mei, Xiajing Zhang, Xuesong Sun, Lihua Hu, Ye Song

**Affiliations:** ^1^Department of Ultrasonography, Shanghai Pudong New Area Zhoupu Hospital, Shanghai University of Medicine and Health Sciences, Shanghai, China; ^2^School of Medical Devices, Shanghai University of Medicine and Health Sciences, Shanghai, China

**Keywords:** sepsis-associated encephalopathy, intensive care unit, transcranial Doppler ultrasonography, cerebral circulation time, contrast-enhanced ultrasound, nomogram

## Abstract

**Background:**

Sepsis-associated encephalopathy (SAE) is prevalent in intensive care unit (ICU) environments but lacks established treatment protocols, necessitating prompt diagnostic methods for early intervention. Traditional symptom-based diagnostics are non-specific and confounded by sedatives, while emerging biomarkers like neuron-specific enolase (NSE) and S100 calcium-binding protein B (S100B) have limited specificity. Transcranial Doppler (TCD) indicators, although is particularly relevant for SAE, requires high operator expertise, limiting its clinical utility.

**Objective:**

This pilot study aims to utilize cerebral circulation time (CCT) assessed via contrast-enhanced ultrasound (CEUS) as an innovative approach to investigate the accuracy of SAE prediction. Further, these CCT measurements are integrated into a nomogram to optimize the predictive performance.

**Methods:**

This study employed a prospective, observational design, enrolling 67 ICU patients diagnosed with sepsis within the initial 24 h. Receiver operating characteristic (ROC) curve analyses were conducted to assess the predictive accuracy of potential markers including NSE, S100B, TCD parameters, and CCT for SAE. A nomogram was constructed via multivariate Logistic Regression to further explore the combined predictive potential of these variables. The model's predictive performance was evaluated through discrimination, calibration, and decision curve analysis (DCA).

**Results:**

SAE manifested at a median of 2 days post-admission in 32 of 67 patients (47.8%), with the remaining 35 sepsis patients constituting the non-SAE group. ROC curves revealed substantial predictive utility for CCT, pulsatility index (PI), and S100B, with CCT emerging as the most efficacious predictor, evidenced by an area under the curve (AUC) of 0.846. Multivariate Logistic Regression identified these markers as independent predictors for SAE, leading to the construction of a nomogram with excellent discrimination, substantiated by an AUC of 0.924 through bootstrap resampling. The model exhibited satisfactory concordance between observed and predicted probabilities, and DCA confirmed its clinical utility for the prompt identification of SAE.

**Conclusion:**

This study highlighted the enhanced predictive value of CCT in SAE detection within ICU settings. A novel nomogram incorporating CCT, PI, and S100B demonstrated robust discrimination, calibration, and clinical utility, solidifying it as a valuable tool for early SAE intervention.

## Introduction

Sepsis-associated encephalopathy (SAE) frequently manifests as cerebral dysfunction in sepsis patients and is often indicative of adverse clinical outcomes. Traditional diagnostic approaches focus on clinical symptoms such as altered consciousness and delirium (1). The reported incidence of SAE varies considerably, ranging from 8% to over 70% among septic patients (2); this variability is partly due to its non-specific diagnostic criteria and frequent underrecognition in medical settings. This diagnostic challenge is exacerbated in intensive care units (ICUs), particularly among sedated and mechanically ventilated patients, as the sedatives confound cognitive assessments (3). Moreover, even when SAE is successfully identified, therapeutic strategies for SAE remain largely unestablished (4). Given these limitations in both diagnosis and treatment, there is an urgent need for prompt and precise diagnostic methods to enable early intervention and potentially improve clinical outcomes.

Emerging diagnostic adjuncts for SAE have attracted clinical attention, including serological biomarkers, electroencephalography (EEG), and transcranial Doppler (TCD) ultrasonography. Serological indicators such as neuron-specific enolase (NSE) and S100 calcium-binding protein B (S100B) offer an objective and cost-effective approach to diagnosis. However, their clinical utility is compromised by suboptimal sensitivity and specificity, making their role in SAE screening and monitoring contentious (2, 5, 6). EEG serves as a sensitive tool for detecting cerebral dysfunction, capable of identifying subclinical SAE and grading its severity. Nevertheless, EEG findings often lack diagnostic specificity for SAE and are further confounded by high variability (7–9). TCD ultrasonography, which non-invasively measures cerebral blood flow velocity, is particularly relevant for SAE given its potential to detect ischemic processes in cerebral circulation (10–12). It holds an advantage over clinical symptom observation and other diagnostic methods by offering real-time data on cerebral hemodynamics. However, this technique is limited by the absence of two-dimensional image guidance and requires a high level of operator expertise.

To address these limitations, this study introduces cerebral circulation time (CCT) assessed via contrast-enhanced ultrasound (CEUS). Similar to TCD, CCT measures the interval between the entry of arterial blood in the internal carotid artery (ICA) and its exit through the internal jugular vein (IJV) (13). It provides multiple advantages, including anatomical visualization and elimination of angle selection inherent in Doppler measurements, thereby enhancing diagnostic accuracy and reliability (14). This is further facilitated in ICU settings by the widespread use of venous catheterization, which simplifies CEUS procedures. While some clinical investigations have touched upon the prognostic implications of CCT in cerebrovascular diseases (15), its prognostic utility specifically in the context of sepsis and its association with SAE has yet to be systematically examined, thereby warranting further investigation.

The primary objective of this pilot study is, firstly, to evaluate the efficacy of CCT as a predictive marker for SAE in the ICU setting. Subsequently, the study will explore the potential of CCT in optimizing the prediction of SAE occurrence, with the aid of a nomogram. By introducing this novel and more reliable assessment tool, this research holds promise for enhancing early intervention strategies and improving clinical outcomes in SAE.

## Materials and methods

This investigation adhered to the ethical principles outlined in the Declaration of Helsinki and obtained approval from the Ethics Committee at Shanghai Pudong New Area Zhoupu Hospital (ZPYYLL-2018-02). Prior to enrollment in the study, written consent was secured from each participant or their legal representatives. Measures were taken to ensure the complete anonymization of all patient-specific data.

### Patient information

To ensure statistical validity, the preliminary step involved the calculation of the required sample size. Guided by Riley et al. (16), it was established that at least ten events per predictor variable were needed to build a multivariate Logistic Regression-based predictive model. Consequently, for a model incorporating at least three predictor variables, a minimum of 30 observed events was required. In light of existing research indicating a 50% incidence of SAE in ICU settings (17, 18), the study needed a minimum of 60 sepsis patients. Subsequent to this calculation, from January 2019 to August 2023, this study enrolled 90 patients admitted to the ICU who were diagnosed with sepsis within the initial 24 h based on the Sepsis-3.0 criteria (19). Inclusion criteria were as follows: (1) age ≥ 18 years, (2) initial admission to the ICU, and (3) an ICU stay of at least 24 h. Exclusion criteria were specified as: (1) primary neurological disorders such as traumatic brain injury or intracranial infections; (2) secondary neurological conditions like hepatic encephalopathy or severe electrolyte imbalance; (3) history of chronic alcohol or drug abuse, dementia, or psychosis; and (4) poor temporal window in TCD. Individuals meeting the eligibility criteria were integrated into the final study cohort.

### Study design

This study employed a prospective, observational design. Upon ICU admission, all sepsis patients were managed according to guideline-based bundle therapy (20). Key demographic, clinical, and laboratory parameters were collected within the initial 24-h period, including but not limited to Glasgow Coma Scale (GCS) scores, Acute Physiology and Chronic Health Evaluation (APACHE) II scores, shock status, levels of NSE and S100B, as well as TCD and CEUS measurements for CCT. Patients with a GCS score below 15 or delirium according to the CAM-ICU checklist, without evidence of direct central nervous system infection, were identified as SAE cases (21). For those diagnosed with SAE upon ICU entry, initial data served for statistical analysis. Conversely, for those not diagnosed with SAE initially, mental status was evaluated twice daily, at 9 a.m. and 5 p.m., by the attending physician until either SAE diagnosis or ICU discharge within a 10-day window. Should a patient be newly diagnosed with SAE during this period, data were updated on that day for statistical purposes. For patients remaining non-SAE, initial data were used for statistical analysis. Mental status assessment for sedated patients was conducted using the Richmond Agitation Sedation Scale (RASS) in conjunction with daily spontaneous awakening trials. A discrepancy of two points or more in the RASS score, as assessed by the attending physician, was considered incongruent with the expected level of sedation. This incongruence, or a patient's failure to awaken within 24 h, was indicative of potential SAE.

### TCD protocol

To minimize operator bias, each TCD assessment was conducted by a physician specifically trained in TCD techniques. Measurements were taken three times through the temporal bone window at both sides of the skull and averaged for accuracy. Within 24 h of ICU admission, subjects underwent TCD evaluation while in a supine position with the bed head leveled. Cerebral hemodynamics were assessed using a 2-MHz ultrasound probe (DWL Doppler-Box; Compumedics). Focusing on the proximal M1 segments of the bilateral middle cerebral arteries, the evaluation recorded key hemodynamic parameters such as systolic peak velocity (Vs), end-diastolic velocity (Vd), mean blood flow velocity (Vm), pulsatility index (PI), and resistive index (RI), specifically from the side of the middle cerebral artery exhibiting higher Vm.

### CEUS protocol for CCT

The CCT measurement was carried out using an EPIQ 7 ultrasonographic scanner (Philips Healthcare, Andover, MA). A high-frequency L4-10 transducer was used for the initial non-contrast assessment to exclude any hemodynamically significant stenoses or anatomical abnormalities in the ICA and IJV, which are critical factors that could potentially influence cerebral blood flow. Utilizing a C5-1 convex array transducer, both the ICA and IJV were visualized in a transverse cross-sectional plane, specifically at a location 1.5 cm superior to the bifurcation of the common carotid artery, to define the area of interest. Upon confirming the target plane, settings were switched to “contrast mode” with reduced mechanical and thermal indices. An FDA-approved microbubble contrast agent (SonoVue, Bracco, Milan, Italy) was prepared in 5 mL of isotonic saline and rapidly administered via the median cubital vein, followed by a 5 mL saline flush. Bolus administration and subsequent CCT assessments were performed on the side demonstrating higher blood flow velocity in earlier TCD measurements.

**Figure 1:**
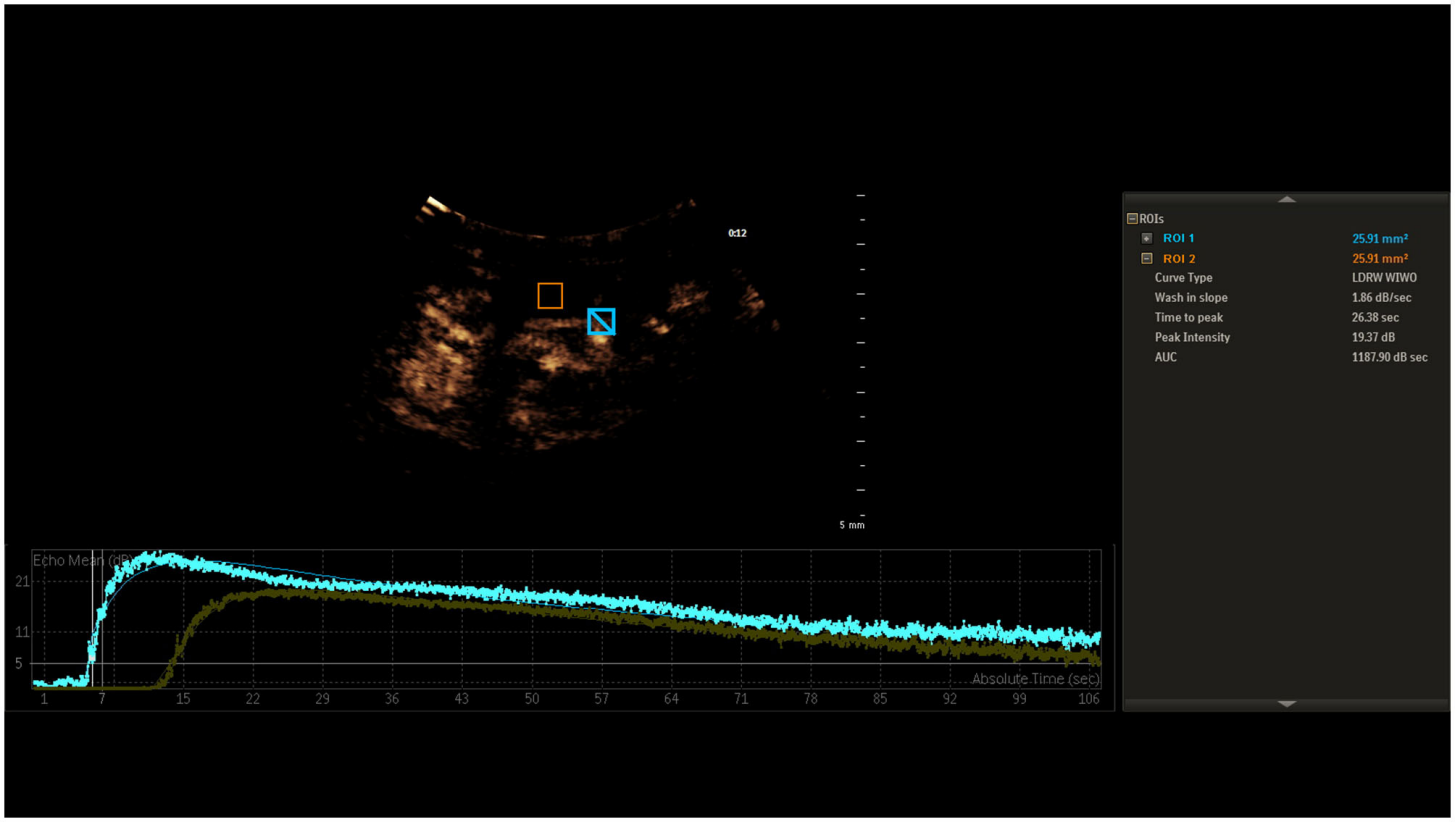
Time-intensity curves for ICA and IJV, highlighting the onset of signal amplification at a 5 dB mean intensity elevation. The interval between these onset points is labeled as CCT.

Analysis of the imaging data was executed through uninterrupted video capture, with time-intensity curves being isolated post-recording by a seasoned ultrasonographer. The in-built software automatically processed these curves after targeting the ICA and IJV, as illustrated in Figure 1. Initial mean intensity for arterial and venous segments was extracted from an early spectrum segment lacking signal boost. A persistent mean intensity elevation of 5 dB or greater over the baseline was earmarked as the onset of signal amplification for both ICA and IJV, based on the methodology suggested by Cavaillon and Chrétien (19). The time difference between these onset points in the ICA and IJV was denoted as CCT.

### Statistical analysis

Statistical evaluations were conducted to distinguish between SAE and non-SAE patients. Normality of continuous variables was assessed by the Shapiro-Wilk test, subsequently guiding the use of either independent-sample *t*-tests or Mann-Whitney *U*-tests. Categorical variables underwent chi-square testing. The accuracy of various predictors was calculated via receiver operating characteristic (ROC) curves and area under the curve (AUC) analyses. Variables with statistically significant differences between patient groups were subjected to multivariate Logistic regression analyses, yielding odds ratios (ORs) and corresponding 95% confidence intervals (CIs). These significant variables were incorporated into the construction of a nomogram designed for the risk assessment of SAE. To assess the robustness of the model, internal validation procedures were applied, augmented by 1,000 iterations of bootstrap resampling to minimize overfitting. Discriminative ability was ascertained through the AUC of the ROC curve, and model calibration was evaluated by both a calibration curve and a Hosmer-Lemeshow goodness-of-fit test. The utility of the model for clinical decision-making was examined using decision curve analysis (DCA). All computational analyses were executed using specialized software: SPSS for statistical evaluations, MedCalc for ROC curve analyses, and the R software package for data visualization.

## Results

### Patient characteristics

**Figure 2:**
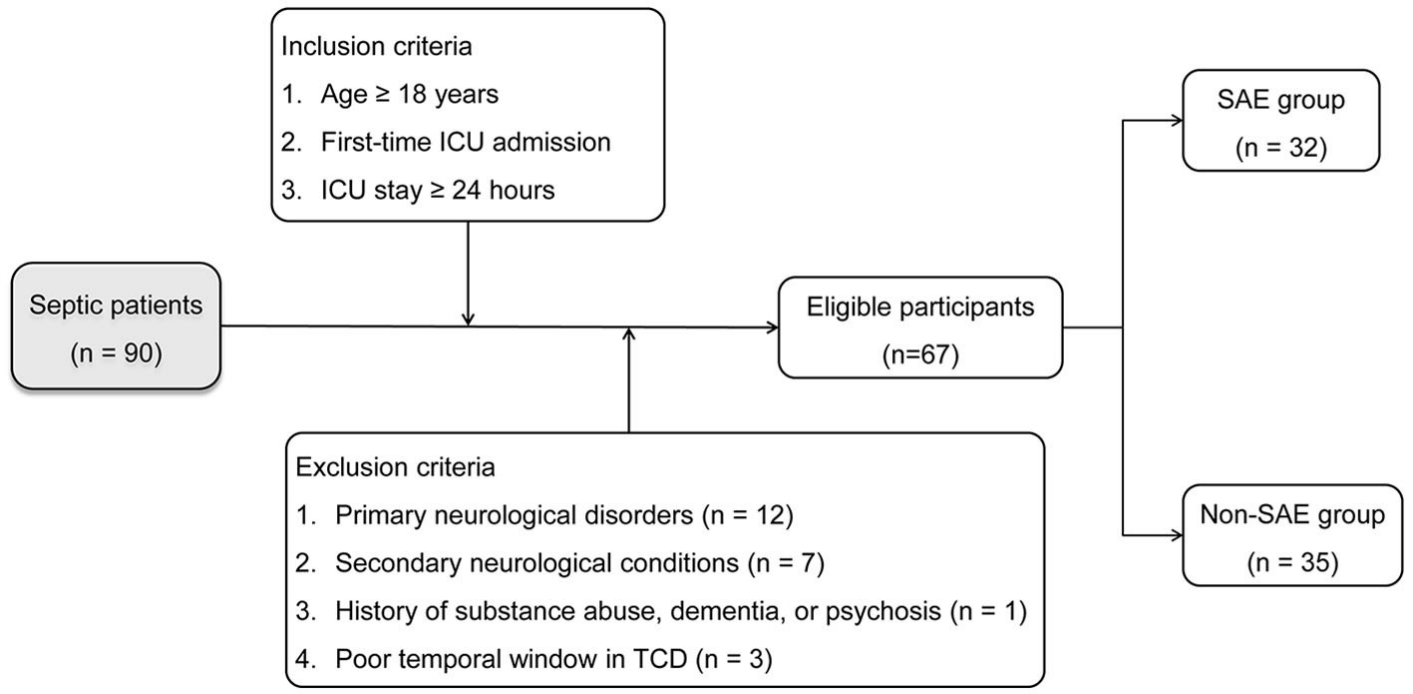
Flowchart illustrating patient selection criteria and allocation into SAE and non-SAE groups.

From the initial cohort of 90 sepsis patients, 12 cases were identified with primary brain injury, seven with secondary brain injury, one with dementia, and three with poor temporal windows rendering TCD parameters unreliable. Subsequently, the remaining 67 patients were selected for a comprehensive analysis. Of these, 32 individuals (47.8%) presented with SAE, and thus constituted the SAE group. The median day of manifestation for SAE was on the 2nd day of admission. The remaining 35 individuals were classified as the non-SAE group for comparative purposes. The process of patient selection is delineated in Figure 2, and Table 1 offers a comparative evaluation of demographic and clinical characteristics between the groups. No statistically significant disparities were observed in age, gender, comorbidity, heart rate, mean arterial pressure (MAP), and peripheral oxygen saturation (SpO2) between the groups, and pulmonary infections emerged as the most prevalent site of infection in both groups. Notably, compared to the non-SAE group, the SAE group exhibited an increased incidence of shock events, had elevated APACHE II scores, and reduced GCS scores. Upon evaluation of potential prognostic indicators, the SAE group displayed significantly elevated levels of NSE, S100B, PI, resistance index (RI), and CCT, whereas values for Vd was notably lower (all *P*-values < 0.05).

**Table 1:** Comparative evaluation of demographic and clinical characteristics between SAE and non-SAE groups.

**Indicator**	**Non-SAE group (*n* = 35)**	**SAE group (*n* = 32)**	***t/χ^2^/Z***	***P***
Age, years	58.83 ± 10.53	57.44 ± 9.55	0.564	0.574
Male, *n* (%)	22 (62.9%)	19 (59.4%)	0.085	0.770
**Comorbidity**, ***n*** **(%)**				
Hypertension	13 (37.1%)	10 (31.3%)	0.257	0.612
Diabetes	7 (20.0%)	6 (18.8%)	0.017	0.897
Heart rate, min^−1^	89.94 ± 14.58	86.16 ± 11.02	1.191	0.238
MAP, mmHg	75.46 ± 13.61	78.66 ± 9.74	1.097	0.277
SpO2, %	97 (96, 99)	97 (96, 98)	0.834	0.404
Shock, *n* (%)	9 (25.7%)	23 (71.9%)	14.276	< 0.001
APACHE II	16 (14–21)	23(20–26)	5.347	< 0.001
GCS	15.0 (14.5–15.0)	11.0 (9.0–12.0)	7.274	< 0.001
NSE, ng/ml	17.09 ± 4.37	19.91 ± 4.93	2.485	0.016
S100B, μg/L	0.12 ± 0.03	0.17 ± 0.04	4.681	< 0.001
Vs, cm/s	96.46 ± 16.19	89.41 ± 16.81	1.748	0.085
Vd, cm/s	41.71 ± 6.49	35.62 ± 8.65	3.277	0.002
Vm, cm/s	61 (53–66)	56 (48–63)	1.929	0.054
PI	1.02 ± 0.11	1.22 ± 0.18	5.512	< 0.001
RI	0.58 (0.53–0.62)	0.63 (0.55–0.69)	2.438	0.015
CCT, s	10.66 ± 1.31	12.70 ± 1.55	5.849	< 0.001
Infection site, *n* (%)			6.184	0.103
Abdominal cavity	11 (31.4%)	9 (28.1%)		
Lung	20 (57.1%)	19 (59.4%)		
Urinary system	2 (5.7%)	2 (6.2%)		
Others	2 (5.7%)	2 (6.2%)		

t, independent-sample *t*-test; χ^2^, chi-square testing; Z, Mann-Whitney U-test.

### Predictive accuracy of potential markers

**Figure 3:**
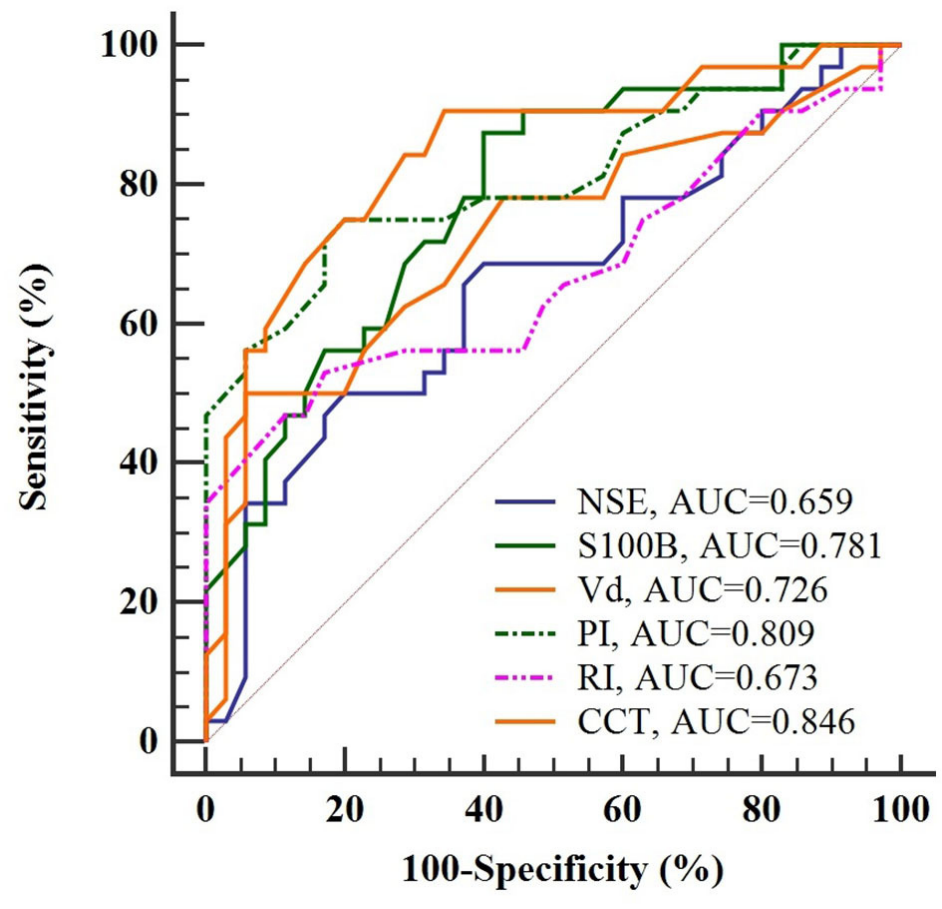
ROC curve analysis of potential indicators for predicting SAE in sepsis patients. CCT emerges as the most accurate indicator with an AUC of 0.846, yet falls short of the 0.9 threshold for independent prediction.

The utility of prospective indicators for predicting SAE was evaluated via ROC curve analyses, graphically represented in Figure 3. A comparison of the AUC values revealed three markers exceeding an AUC of 0.75, indicative of substantial predictive utility. Specifically, CCT emerged as the most efficacious predictor for SAE, with an AUC of 0.846; however, it did not meet the more stringent criterion of an AUC of 0.9. Following closely were PI and S100B, with AUC values of 0.809 and 0.781, respectively. These findings suggested that, although these markers manifested significant prognostic capabilities, none could serve as an isolated, robust predictor for the onset of SAE.

### Nomogram for SAE risk assessment

**Figure 4:**
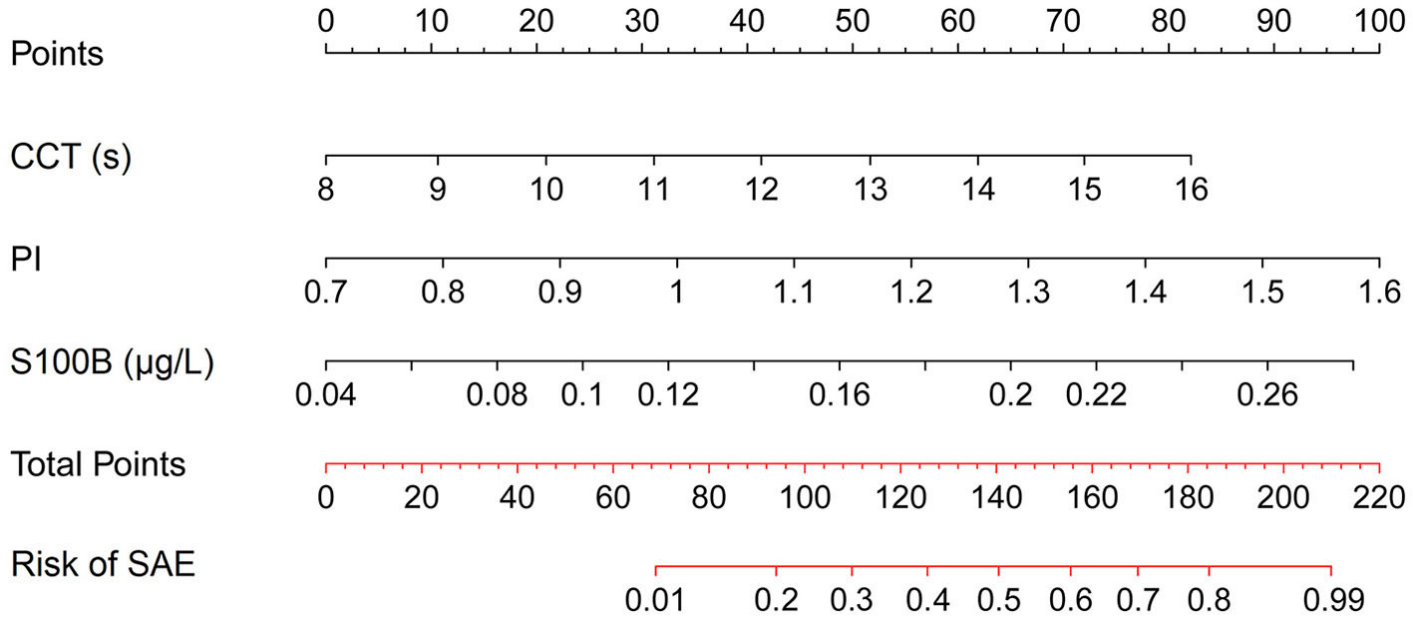
Nomogram for estimating the likelihood of SAE based on selected prognostic markers.

To assess the feasibility of a combined predictive approach, multivariate Logistic Regression was applied to evaluate a set of relevant prognostic markers, as detailed in Table 2. S100B, PI, and CCT were identified as the independent predictors for SAE. Utilizing the OR values as a basis for weighting, a nomogram was developed to illustrate the probabilistic incidence of SAE. Figure 4 provides a graphical representation of this model, wherein the estimated probability of SAE could be derived by summing the scaled points associated with each variable. The cumulative score then corresponded to the risk gradient indicated on the lower axis.

**Table 2:** Multivariate logistic regression analysis of independent predictors for SAE.

**Indicator**	***B***	**S.E**.	**Wald**	***P***	**OR**	**95% CI**
NSE	−0.005	0.132	0.002	0.968	0.995	0.768–1.288
S100B	0.497	0.215	5.321	0.021	1.643^*^	1.078–2.505
Vd	−0.112	0.065	2.994	0.084	0.894	0.787–1.015
PI	0.154	0.058	6.965	0.008	1.166^*^	1.040–1.307
RI	0.069	0.084	0.679	0.410	1.071^*^	0.909–1.262
CCT	1.090	0.453	5.775	0.016	2.974	1.223–7.232

The OR values denoted by ^*^ indicate the increased risk associated with each 0.01-unit increment.

### Model performance validation

**Figure 5:**
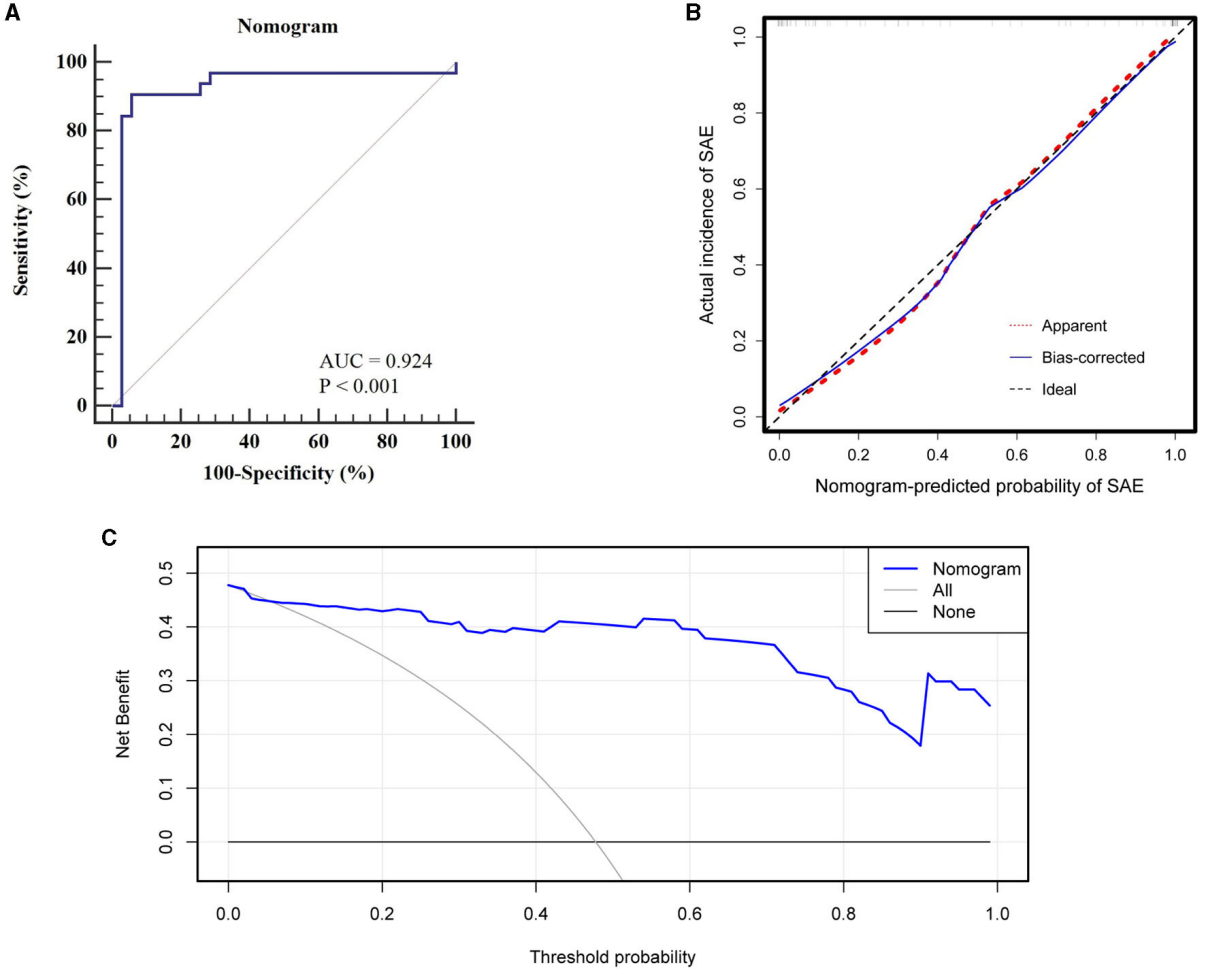
Evaluation of nomogram predictive accuracy: **(A)** ROC curve with an AUC of 0.924 for discriminatory capacity, **(B)** Calibration curve indicating high concordance between observed and predicted probabilities, **(C)** DCA showing net clinical benefit across a 0–100% threshold probability range.

Figure 5 offers an evaluation of the predictive accuracy inherent in the nomogram. Through 1,000 bootstrap resamples, an AUC of 0.924 (95% CI, 0.833–0.975) was achieved, substantiating the superior discriminatory capacity of the model. Both a calibration plot and a Hosmer-Lemeshow test corroborated the concordance between observed and predicted probabilities (χ^2^ = 2.840, *P* = 0.944). DCA further emphasized the clinical utility of the model by indicating net benefit across a threshold probability range of 0–100%.

## Discussion

To the best of our knowledge, this study serves as a seminal evaluation of CCT as a reliable prognostic indicator for SAE within the critical care environment. Through ROC curve analyses, we comparatively assessed the predictive capabilities of multiple clinical indices, highlighting the superior efficacy of CCT in SAE anticipation. Utilizing multivariate Logistic Regression, an integrated nomogram was formulated, incorporating salient indicators such as CCT, S100B, and PI, thereby emerging as a robust diagnostic tool for the clinical management of sepsis. In comparison to conventional symptom-oriented evaluations, this model enhances prediction objectivity and minimizes susceptibility to confounding variables, thus offering clinical utility for the prompt identification and intervention of SAE in sepsis patients.

CCT, an essential parameter for assessing cerebral hemodynamics, has been traditionally utilized in the evaluation of vascular pathologies like arteriovenous malformations and neurodegenerative diseases such as Alzheimer's (14, 15, 22). However, its applicability for SAE prediction in ICU settings remains underexplored. This study serves as a novel investigation into the predictive utility of CCT, as measured by CEUS, for SAE in sepsis patients. Conventional techniques, such as TCD ultrasonography, have been limited by high operator dependency, restricting their clinical application (13, 23). CEUS, in contrast, affords real-time, two-dimensional imaging of cerebral vasculature and perfusion, thereby overcoming operator-dependent limitations and streamlining diagnosis (24). Additionally, CEUS provides a more objective assessment of cerebral microcirculatory changes, minimizing diagnostic difficulties often associated with interventions like mechanical ventilation or sedative administration. This advantage becomes even more pronounced in the context of ICU settings, where venous catheterization is routinely performed, thereby rendering the venous introduction of contrast agents both feasible and convenient. For precise CCT measurements, maintaining transducer cross-sectional stability with consistent, minimal pressure is essential. Empirical data suggest that variations in pressure can deform the IJV, while shifts in the imaging plane may introduce variability into the contrast agent distribution curve, thereby compromising result reliability.

In this study, CCT exhibited superior predictive validity for SAE over PI and S100B, as evidenced by a higher AUC. However, the suboptimal AUC of CCT highlighted its limitations in risk stratification if used as a solitary diagnostic modality. Given the comparable ROC curve distributions for CCT, PI, and S100B and their less-than-ideal specificity for predicting SAE, a combined predictive model was formulated to augment collective predictive specificity. The multivariable Logistic Regression analysis revealed CCT, PI, and S100B as independent prognostic indicators for SAE. Beyond CCT, the diagnostic utility of PI has been extensively examined; it serves as a consistent gauge of cerebral vascular resistance and shows a direct relationship with peripheral vascular resistance variations (25). Elevated PI levels have been implicated in cognitive dysfunction in patients with sepsis (26). Concurrently, high expression levels of S100B are involved in neuroinflammation and have been notably associated with neurodegenerative disorders such as Alzheimer's disease and Parkinson's disease (27, 28). Importantly, an animal study suggested that S100B emanated primarily from the brain during endotoxemia and played a pivotal role in acute brain injury and long-term cognitive impairment (29). These insights underscore the potential of S100B as an integral biomarker for SAE.

To enhance clinical utility, a nomogram was constructed for visual representation of the combined predictive model. Upon validation, the model demonstrated a discriminatory capacity exceeding the threshold of 0.9, thereby surpassing models previously established by Lu et al. (18) and Zhao et al. (30), which relied on traditional clinical indicators. This superior performance is attributed to the enhanced predictive specificity achieved by the integration of CCT, PI, and S100B as composite indicators. Moreover, the model's calibration and DCA curves revealed commendable predictive consistency and clinical applicability, suggesting its potential for early identification of SAE. In practical terms, ICU clinicians can employ this nomogram to calculate the risk of SAE occurrence for individual patients. For instance, a sepsis patient with CCT, PI, and S100B values of 13 s, 1.3, and 0.18 μg/L, respectively, would accumulate a nomogram score of ~170, which translates to an estimated 70% likelihood of SAE development. Such a predictive tool enables the initiation of targeted interventions to avert the onset of SAE prior to the manifestation of overt cognitive alterations.

Certain limitations in the present study warrant discussion. Conducted as a single-center, prospective investigation with a confined patient cohort, the study may face challenges in broader applicability of the developed nomogram. As an evidence-based pilot study, additional potential confounding factors may have not been considered and discussed, warranting further exploration in future research. Although short-acting sedatives were administered, the 24-h period may not allow for complete drug clearance in some individuals, and sedation itself could induce delirium, complicating the assessment of SAE. Moreover, a 3-day stay in the ICU could induce cognitive disturbances even without sepsis, adding complexity to the differentiation between such states and SAE. Due to the limited sample size, only internal validation was performed, lacking external corroboration from additional centers. Despite these constraints, the study highlights the potential utility of the nomogram in SAE prediction and underscores the need for future multi-center, prospective research to enhance model reliability and clinical relevance.

In conclusion, this study revealed the superior predictive capability of CCT over other potential predictors in the assessment of SAE within intensive care environments. To further enhance this predictive accuracy, the study introduced an innovative nomogram incorporating CCT, PI, and S100B. The model demonstrated robust discrimination, calibration, and clinical utility, offering a valuable tool for early intervention strategies for SAE.

## Data availability statement

The raw data supporting the conclusions of this article will be made available by the authors, without undue reservation.

## Ethics statement

The studies involving humans were approved by the Institutional Review Board of Shanghai Pudong New Area Zhoupu Hospital. The studies were conducted in accordance with the local legislation and institutional requirements. Written informed consent to participate in this study was provided by the patients/participants or patient/participants' legal guardian/next of kin.

## Author contributions

JM: Conceptualization, Data curation, Formal analysis, Funding acquisition, Investigation, Methodology, Software, Validation, Visualization, Writing – original draft. XZ: Conceptualization, Data curation, Resources, Software, Supervision, Validation, Writing – original draft. XS: Conceptualization, Data curation, Formal analysis, Investigation, Project administration, Writing – original draft. LH: Data curation, Investigation, Resources, Writing – original draft. YS: Conceptualization, Formal analysis, Funding acquisition, Investigation, Project administration, Resources, Supervision, Validation, Visualization, Writing – review & editing.
